# Disseminated *Spiroplasma apis* Infection in Patient with Agammaglobulinemia, France

**DOI:** 10.3201/eid2412.180567

**Published:** 2018-12

**Authors:** Nicolas Etienne, Laurent Bret, Cécile Le Brun, Hervé Lecuyer, Josquin Moraly, Fanny Lanternier, Olivier Hermine, Agnès Ferroni, Marc Lecuit, Sabine Pereyre, Laure Beven, Olivier Lortholary

**Affiliations:** Paris Descartes University Hospital, Paris, France (N. Etienne, H. Lecuyer, F. Lanternier, O. Hermine, A. Ferroni, M. Lecuit, O. Lortholary);; Centre Hospitalier Régional Orléans, France (L. Bret);; Centre Hospitalier Régional, Universitaire, Tours, France (C. Le Brun); Pasteur Institute, Paris (M. Lecuit);; University of Bordeaux, Bordeaux, France (S. Pereyre);; University of Bordeaux, Villenave d’Ornon, France (L. Beven)

**Keywords:** *Spiroplasma*, *Spiroplasma apis*, agammaglobulinemia, immunocompromised, Bruton, endocarditis, bacterial endocarditis, infective endocarditis, septic arthritis, bacterial infection, x linked agammaglobulinemia, hypogammaglobulinemia, genetic diseases, btk, bruton thyrosine kinase, immunocompromised patient, mycoplasma, insect stings, bee, hornet, *Apis*, *Apis mellifera*, bacteria, vector-borne infections, France

## Abstract

We report a disseminated infection caused by *Spiroplasma apis*, a honeybee pathogen, in a patient in France who had X-linked agammaglobulinemia. Identification was challenging because initial bacterial cultures and direct examination by Gram staining were negative. Unexplained sepsis in patients with agammaglobulinemia warrants specific investigation to identify fastidious bacteria such as *Spiroplasma* spp.

In February 2017, a 40-year-old man in France who was under immunoglobulin replacement therapy for X-linked agammaglobulinemia experienced a migrating nonitchy papular eruption. Skin biopsy revealed nonspecific lymphocytic dermatitis. One month later, he experienced distal interphalangeal arthritis. Histology of a synovial biopsy sample found a nonspecific lymphocytic infiltrate. Bacterial cultures were negative. In April 2017, febrile arthritis of the left ankle and right knee appeared, along with bilateral wrist and finger tenosynovitis (Technical Appendix Figure). We initiated ceftriaxone (1 g/d) and performed a left ankle joint aspiration 24 hours later. The patient remained febrile with a pulmonary valve murmur of tricuspid regurgitation and purpuric lesions, tenosynovitis in the left ankle and both wrists, and right knee and interphalangeal polyarthritis. Serum C-reactive protein was 136 mg/dL and IgG serum level 9.4 g/L. Transesophageal echography showed slight tricuspid regurgitation. Arthritis worsened, and we performed a right knee joint aspiration. Four of 6 blood cultures were positive after 55 h of incubation at 37°C (Bactec FX, Becton Dickinson, Franklin Lakes, NJ, USA). Results of direct examination by Gram staining of positive blood cultures and real-time multiplex PCR for blood culture identification (FilmArray BCID Panel; bioMérieux, Marcy l’Etoile, France) were negative. Small α-hemolytic areas appeared on subcultures in horse blood agar after 5 days of aerobic culture; results of mass spectrometry performed on these areas were negative. Blood culture–specific PCR targeting *Tropheryma whipplei*, *Bartonella* spp., *Brucella* spp., *Coxiella burnetii,* and *Rickettsia* spp. showed negative results.

At this stage, we modified antimicrobial therapy to be optimal for *Mycoplasma*, *Chlamydia*, deficient streptococci, *Campylobacter,* and *Helicobacter* regarding the context of agammaglobulinemia. We introduced amoxicillin/clavulanic acid (12 g/1,200 mg/d) associated with levofloxacin (500 mg 2×/d), leading to apyrexia and negative blood culture after 1 day. Results of testing for *Chlamydia trachomatis* (by PCR and culture) and *Ureaplasma* spp. and *Mycoplasma hominis* (by culture) in urine were negative. We seeded synovial fluid on blood culture, and small colonies developed on horse blood agar. We extracted DNA from the zone of hemolysis using EZ1 DNA tissue kit (QIAGEN, Valencia, CA, USA). We amplified and sequenced the 16S rRNA gene with the primer set 27F/16S1RRB as previously described (*1*) and performed bacterial identification using phylogenic analysis on the Bioinformatics Bacterial Identification tool (*2*). The 491-bp amplicon sequence was 100% identical to that of *Spiroplasma apis* strain ATCC 33834 (GenBank accession no. GU993267). We confirmed identification of *S. apis* by specific *S. apis* PCR (*3*) and culture (on modified SP4 agar) in synovial fluid from left ankle and right knee and in blood culture. Direct examination of synovial fluid and blood culture with dark field microscopy allowed identification of bacteria with a helical morphology and motility evocative of spiroplasmas (Figure). We obtained highly enriched *Spiroplasma* cultures starting from fluid and blood cultures. We retrospectively found specific PCR for *S. apis* positive in synovial biopsy from the distal interphalangeal sample. Antimicrobial drug susceptibility testing showed susceptibility to macrolides, tetracyclines, and fluoroquinolones and resistance to rifampin, cotrimoxazole, and penicillin G. Tetracycline and fluoroquinolone MIC values were similar to those reported for *Ureaplasma* spp.

**Figure:**
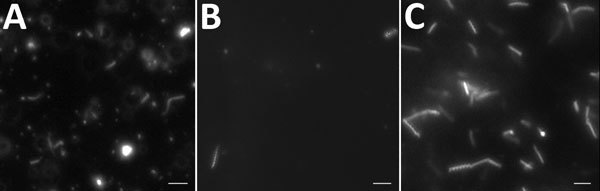
Direct examination with dark-field microscopy of specimens from a patient with agammaglobulinemia who had *Spiroplasma apis* infection, France. A) Helical and motile bacteria in blood culture. B) Elongated and coccoid bacteria in joint fluid. C) Helical and motile bacteria in culture from joint fluid in modified SP4 broth medium. Scale bar indicates 10 µm.

Patient’s condition gradually improved with 6-week administration of levofloxacin and iterative joint aspirations of the right knee. Because of severe tendinopathy, levofloxacin was stopped after 6 weeks and replaced by azithromycin (500 mg/d) and doxycycline (200 mg 2×/d) for 6 weeks.

Patients with agammaglobulinemia are susceptible to infections, including mycoplasma infections (*4*). Invasive infections such as endocarditis and septic arthritis have been reported with *M. hominis* and *Ureaplasma urealyticum* in hypogammaglobulinemic patients. Of note, our patient contracted mycoplasma infection despite an appropriate gammaglobulin serum level (IgG 9.4 g/L).

Spiroplasmas, arthropod-infecting bacteria of the class Mollicutes, are similar to mycoplasmas. Pathogenic species include *S. apis*, detected in hives in southwestern France (*5*). *S. apis* is associated with the lethal May disease of the European honeybee *Apis mellifera*. Its prevalence in bee colonies in France was not reported but was recently shown to reach high percentages in other countries (*6*). Only 3 cases of human infection due to *Spiroplasma* spp. have been reported: hepatitis in a lung transplant recipient (*7*), uveitis in a premature neonate (*8*), and bacteremia in a patient under certolizumab treatment (*9*). None of these infections correlated with any noticeable insect bite history. In contrast, the patient we report had multiple stings by a flying insect resembling a hornet in August 2016, in the Loiret department of France, leading to cutaneous blebs and transitory rash. This finding raises the possibility that *S. apis* might infect insects other than honeybees. The insect stings in this patient are a likely gateway of the reported infection.

In summary, clinicians and microbiologists should be aware of fastidious organisms in atypical infections in immunocompromised patients. Our findings indicate a need for prolonged culture on specific agar on all joint fluids in patients with agammaglobulinemia and targeted molecular methods to identify *S. apis* organisms.

Technical Appendix Additional information about *Spiroplasma apis *infection in a patient with agammaglobulinemia, France.

## References

[1] Schabereiter-Gurtner C, Nehr M, Apfalter P, Makristathis A, Rotter ML, Hirschl AM. Evaluation of a protocol for molecular broad-range diagnosis of culture-negative bacterial infections in clinical routine diagnosis. J Appl Microbiol. 2008;104:1228–37. 10.1111/j.1365-2672.2007.03648.x 18028360

[2] Devulder G, Perrière G, Baty F, Flandrois JP. BIBI, a bioinformatics bacterial identification tool. J Clin Microbiol. 2003;41:1785–7. 10.1128/JCM.41.4.1785-1787.2003 12682188 PMC153906

[3] Meeus I, Vercruysse V, Smagghe G. Molecular detection of Spiroplasma apis and Spiroplasma melliferum in bees. J Invertebr Pathol. 2012;109:172–4. 10.1016/j.jip.2011.11.006 22138255

[4] Blanchard A, Bébéar CM. Mycoplasmas of humans. In: Razin S, Herrmann R, editors. Molecular biology and pathogenicity of mycoplasmas. New York: Kluwer Academic/Plenum Publishers; 2002. p. 45–71.

[5] Mouches C, Bové JM, Tully JG, Rose DL, McCoy RE, Carle-Junca P, et al. Spiroplasma apis, a new species from the honey-bee Apis mellifera. Ann Microbiol (Paris). 1983;134A:383–97. 6195951

[6] Schwarz RS, Teixeira ÉW, Tauber JP, Birke JM, Martins MF, Fonseca I, et al. Honey bee colonies act as reservoirs for two Spiroplasma facultative symbionts and incur complex, multiyear infection dynamics. MicrobiologyOpen. 2014;3:341–55. 10.1002/mbo3.172 24771723 PMC4082708

[7] Mueller NJ, Tini GM, Weber A, Gaspert A, Husmann L, Bloemberg G, et al. Hepatitis from Spiroplasma sp. in an immunocompromised patient. Am J Transplant. 2015;15:2511–6. 10.1111/ajt.13254 25832127

[8] Lorenz B, Schroeder J, Reischl U. First evidence of an endogenous Spiroplasma sp. infection in humans manifesting as unilateral cataract associated with anterior uveitis in a premature baby. Graefes Arch Clin Exp Ophthalmol. 2002;240:348–53. 10.1007/s00417-002-0453-3 12073057

[9] Aquilino A, Masiá M, López P, Galiana AJ, Tovar J, Andrés M, et al. First human systemic infection caused by Spiroplasma. J Clin Microbiol. 2015;53:719–21. 10.1128/JCM.02841-14 25428150 PMC4298541

