# Supplementary material for: Disseminated Spiroplasma apis Infection in Patient with Agammaglobulinemia, France
Source: Emerg Infect Dis. 2018 Dec;24(12):2382–4. doi: 10.3201/eid2412.180567 (PMC6256403; doi:10.3201/eid2412.180567)
Supplement: Technical Appendix — Additional information about Spiroplasma apis infection in a patient with agammaglobulinemia, France. [file 18-0567-Techapp-s1.pdf]

Article DOI: https://doi.org/10.3201/eid2412.180567

Disseminated *Spiroplasma apis* Infection in Patient with Agammaglobulinemia, France

Technical Appendix

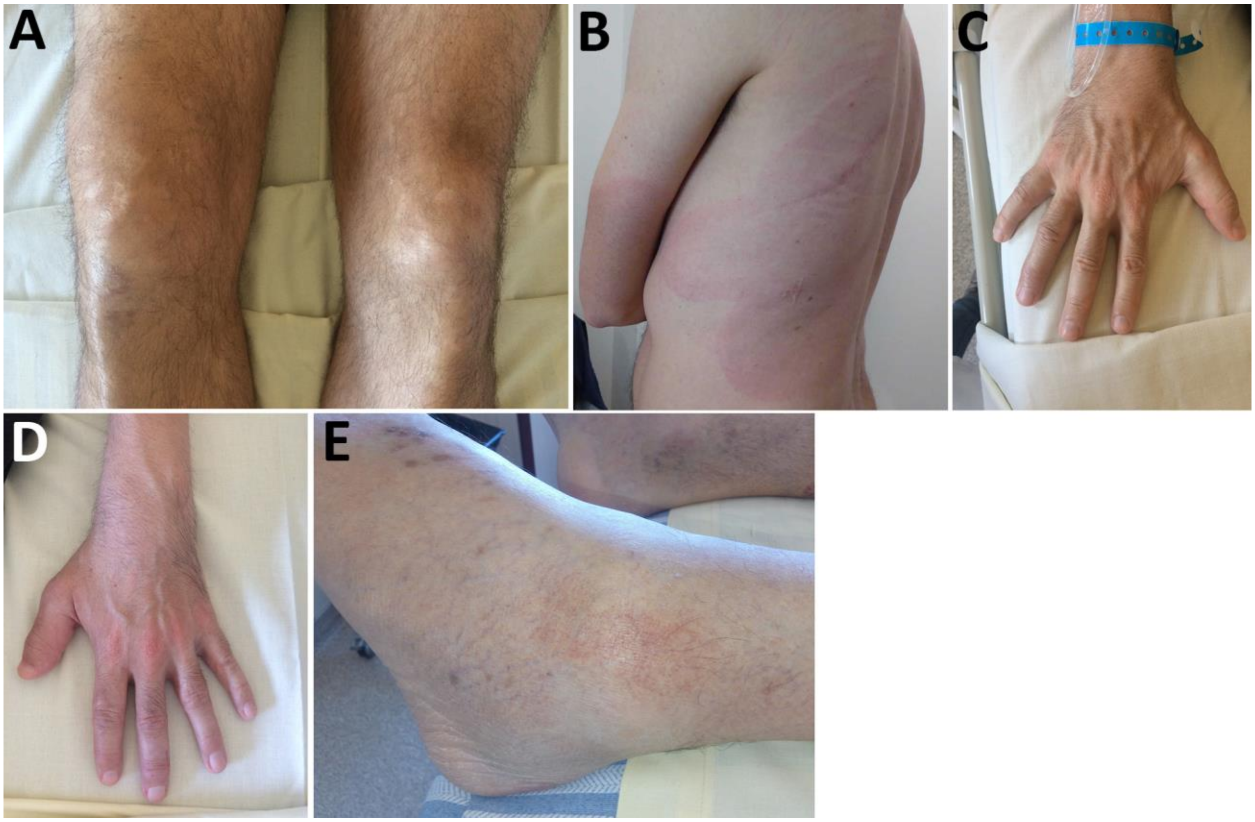

Technical Appendix Figure. Clinical symptoms of *Spiroplasma apis* infection in a patient. A) Arthritis in knee. B) Erythema. C) Tenosynovitis in right wrist. D) Tenosynovitis in left wrist. E) Arthritis in left ankle.
